# Levomethadone Selectively Reduces Emotional Impulsivity in ASRS-Positive ADHD–OUD Patients, Independent of Dose Escalation

**DOI:** 10.3390/jcm15010089

**Published:** 2025-12-23

**Authors:** Alessandro Pallucchini, Maurizio Varese, Irene Pergentini, Elisa Cerrai, Samuele Gemignani, Elisa Parapetto, Francesco Simonetti, Icro Maremmani, Angelo G. I. Maremmani

**Affiliations:** 1 Department of Psychiatry and Addictions, Section of Addictions, North-Western Tuscany Local Health Unit, Tuscany NHS, Apuan Zone, Via Carriona, 241/B, 54033 Carrara, Italy; maurizio.varese@uslnordovest.toscana.it (M.V.); samuele.gemignani@uslnordovest.toscana.it (S.G.); 2 Department of Psychiatry and Addictions, Section of Addictions, North-Western Tuscany Local Health Unit, Tuscany NHS, Lucca Zone, Cittadella della Salute “Ospedale Campo di Marte”, 55100 Lucca, Italy; irene.pergentini@uslnordovest.toscana.it (I.P.); elisa.cerrai@uslnordovest.toscana.it (E.C.); 3 Department of Psychiatry and Addictions, Section of Addictions, North-Western Tuscany Local Health Unit, Tuscany NHS, Livorno Zone, Via Tiberio Scali, 11, 57121 Livorno, Italy; elisa.parapetto@uslnordovest.toscana.it; 4 Department of Psychiatry and Addictions, Section of Addictions, North-Western Tuscany Local Health Unit, Tuscany NHS, Viareggio Zone, Via della Gronda, 15, 55049 Viareggio, Italy; francesco.simonetti@uslnordovest.toscana.it; 5 VP Dole Research Group, G. De Lisio Institute of Behavioral Sciences, Via di Pratale 3, 56121 Pisa, Italy; icromaremmani@gmail.com (I.M.); angelogiovanniicro.maremmani@unicamillus.org (A.G.I.M.); 6 Department of Medicine, Saint Camillus International University of Health Sciences, Via di Sant’Alessandro 8, 00131 Rome, Italy

**Keywords:** ADHD, emotional dysregulation, impulsivity, levomethadone, linear mixed model, opioid use disorder, substance use

## Abstract

**Background:** Emotional dysregulation and impulsivity represent key risk factors for adverse trajectories in adults with ADHD and are frequently observed among patients with opioid use disorder (OUD). Levomethadone, the R-enantiomer of methadone, provides more stable dopaminergic modulation than the racemic formulation and may improve emotional control. The primary objective was to examine emotional, clinical, and substance use changes after the switch to levomethadone and to determine whether these trajectories differed according to ADHD screening status. This study evaluated emotional, clinical, and behavioral outcomes—including substance use—after transitioning from racemic methadone to levomethadone maintenance therapy, focusing on the moderating role of ADHD symptoms and dose escalation. **Methods:** Eighty-three OUD patients in methadone maintenance were assessed at baseline, T1 (mean = 2.13 months, SD = 0.65), and T2 (mean = 6.20 months, SD = 0.91). Emotional dysregulation (RIPOST), clinical severity (Clinical Global Impression), and days of substance use were analyzed using Linear Mixed Models (participants with ≥1 valid follow-up). ADHD symptoms (Adult ADHD Self-Report Scale DSM-5) were evaluated with Wilcoxon signed-rank tests. Dose escalation (↑levomethadone) was defined as ≥1 increase during follow-up and was only included in the mixed models. Substance use analyses were restricted to baseline active users. **Results:** Emotional impulsivity significantly decreased over time only in participants screening positive for ADHD symptoms (ASRS ≥ 14), independent of dose escalation. Emotional instability also declined but across the full cohort. CGI scores improved in all participants. Substance use patterns showed a modest overall improvement, with reductions most evident for sedatives and alcohol. The findings indicate a specific effect of levomethadone on affective regulation and clinical stabilization, particularly in individuals with impulsivity traits. **Conclusions:** Levomethadone maintenance appears to improve emotional regulation and global functioning beyond dose-related effects, supporting its potential value in complex OUD patients with clinically relevant ADHD symptomatology. Combined treatment with levomethadone and methylphenidate may further enhance executive control and craving regulation in this population.

## 1. Introduction

Attention-Deficit/Hyperactivity Disorder (ADHD) is highly prevalent among individuals receiving treatment for substance use disorders (SUDs). Epidemiological evidence suggests that ADHD affects approximately 23% of patients treated in addiction services [1,2], with rates reaching up to 33% among individuals with opioid use disorder (OUD) [3], a figure markedly higher than that observed in the general adult population, where prevalence is estimated at approximately 2.5–5% [4].

Individuals with ADHD display elevated rates of alcohol, stimulant, opioid, and cannabis use [5,6]. Importantly, adolescents with untreated ADHD are at a substantially higher risk of developing SUDs later in life compared with non-ADHD peers [7,8]. This vulnerability has been linked primarily to executive function deficits, impulse control difficulties, and emotional dysregulation [9,10], which contribute to maladaptive coping behaviors and addictive trajectories.

Nevertheless, when ADHD co-occurs with opioid use disorder (OUD), the clinical presentation tends to be more severe [11]. These patients often exhibit marked impulsivity and negative urgency defined as rash action under negative affect [12,13].

Emotional dysregulation in adults with ADHD has been associated with increased relapse risk and impaired adherence; however, these vulnerabilities do not inherently predict poorer treatment retention when adequately managed. Indeed, a five-year longitudinal study showed that adults with ADHD, regardless of comorbid SUD, had comparable retention rates when receiving stimulant or non-stimulant therapies targeting ADHD symptoms [14]. Levomethadone, the levo-rotatory R-enantiomer of methadone, acts as a full μ-opioid receptor (MOR) agonist with high affinity and efficacy, selectively activating MORs co-expressed with the galanin-1 receptor (Gal_1_R) in the ventral tegmental area (VTA). This MOR–Gal_1_R heteromer is a receptor complex crucial for the dopaminergic and reinforcing effects of opioids, and its activation by R-methadone enhances dopaminergic firing and dopamine release in mesolimbic pathways [15].

Notably, R-methadone displays a dose-dependent inverted U-shaped response profile, whereby its reinforcing and locomotor-activating effects increase at low-to-moderate doses, but plateau at higher doses. This non-linear pattern has been linked to MOR saturation, acute receptor desensitization, and the recruitment of anti-reward mechanisms that limit dopaminergic output under high agonist exposure [16,17,18]. In contrast, the S-enantiomer (S-methadone or es-methadone) shows markedly lower intrinsic efficacy at MORs and fails to activate the MOR–Gal_1_R heteromer. Experimental evidence demonstrates that S-methadone instead acts as a functional antagonist at this receptor complex: at higher doses, it dose-dependently suppresses the dopaminergic and locomotor-stimulating effects of R-methadone, ultimately blocking R-induced dopamine release in the VTA [19]. A substantial body of evidence indicates that dysregulation of the dopaminergic system underlies the core symptoms of ADHD. Altered signaling in mesocorticolimbic dopamine pathways contributes to impaired executive control, increased impulsivity, and heightened sensitivity to reward delay [20,21].

This mechanism is supported by neuroimaging findings and by the clinical efficacy of stimulant medications such as methylphenidate, which enhance synaptic dopamine and norepinephrine levels [22]. These findings suggest that levomethadone’s pharmacodynamic profile may hold particular clinical relevance for ADHD patients, even though its pro-dopaminergic effect does not increase linearly with dosage or over time [23].

To date, no study has examined emotional or clinical changes associated with switching from racemic methadone to levomethadone in patients screened for ADHD symptoms. This gap is clinically relevant, as ADHD-related emotional dysregulation and impulsivity may interact with dopaminergic adaptations occurring during long-term opioid agonist therapy. Neuroimaging evidence indicates that chronic racemic methadone administration is associated with reduced striatal dopamine transporter availability and diminished dopaminergic function [24], potentially contributing to motivational blunting and poorer clinical outcomes in vulnerable patients, including those with ADHD. Although emotional dysregulation is a well-established determinant of addiction outcomes [25,26,27], its specific dimensions have rarely been examined longitudinally in ADHD–SUD comorbidity during opioid agonist treatment.

The present study examined a clinical cohort undergoing transition from racemic methadone to levomethadone, comparing patients with and without ADHD-positive screening. Specifically, we examined the longitudinal trajectories of emotional dysregulation, clinical severity, and substance use frequency after the transition to levomethadone; the role of dose escalation on these outcomes; and whether ADHD symptomatology moderated these clinical and behavioral changes over time.

We hypothesized that participants with a positive ADHD screening would show greater emotional dysregulation over time; that levomethadone would moderate these trajectories; and that dose escalation might influence emotional control, clinical severity, and substance use frequency among baseline active users.

## 2. Materials and Methods

### 2.1. Study Design and Participants

This study employed an observational longitudinal design with three assessment points, baseline, T1 (mean = 2.13 months, SD = 0.65), and T2 (mean = 6.20 months, SD = 0.91), to evaluate emotional dysregulation, clinical outcomes (CGI), and substance use frequency following the switch from racemic methadone to levomethadone maintenance therapy. The analyses also examined the impact of levomethadone dose escalation defined as at least one escalation during the follow-up period on both clinical and substance use outcomes. Participants were included in the Linear Mixed Models only if they had at least one valid follow-up observation for the corresponding outcome. Substance use analyses were further restricted to baseline active users (i.e., participants reporting substance use at baseline).

Participants were recruited from the outpatient Addiction Services of the North-Western Tuscany Local Health Unit (Italy), encompassing multiple public opioid agonist treatment centers within the regional network. Recruitment and data collection were conducted between 2022 and 2025. All patients were receiving racemic methadone maintenance therapy as part of routine clinical care and were transitioned to levomethadone under medical supervision.

A total of 83 participants completed the baseline assessment. For the clinical outcome scales (RIPOST scales, Clinical Global Impression [CGI], and Adult ADHD Self-Report Scale [ASRS-5]), 71 participants had at least one valid follow-up (“entrants”) and were therefore included in the Linear Mixed Models (LMMs).

For substance use outcomes, analyses were restricted to participants with active use at baseline and at least one valid follow-up, resulting in 30 baseline users (25 entrants) for opioids, 34 (28) for alcohol/sedatives, 38 (32) for stimulants, and 26 (23) for cannabis (THC) (see Table 1).

Follow-up data were available for approximately 75% of participants at T1 and 59% at T2 for RIPOST and CGI, and between 68–77% (T1) and 47–66% (T2) for substance use outcomes, depending on the substance. These proportions indicate an adequate level of follow-up completeness both for clinical and substance use measures, consistent with retention rates typically reported in real-world longitudinal studies of opioid-agonist treatment [28,29].

Inclusion criteria were as follows: age above 18 years; a diagnosis of opioid use disorder according to DSM-5-TR criteria; maintenance treatment with racemic methadone for at least six months before the switch; the ability to provide informed consent; and a stable psychopharmacological regimen for at least six months prior to enrolment and maintained throughout follow-up.

Exclusion criteria were as follows: age below 18 years; the presence of intellectual disability or severe cognitive impairment; and the inability or refusal to provide informed consent.

At the time of the switch, the initial levomethadone dose was calculated as half of the previous racemic methadone dose, consistent with established clinical conversion protocols.

Urine toxicology screening was planned at least twice per week; however, in routine clinical practice the actual frequency could vary, and when not feasible, testing occurred whenever the patient presented for supervised medication administration. Substance use was assessed for opioids, stimulants, cannabis (THC), and alcohol/sedatives using urine toxicology results and self-reports corroborated by family members or clinical staff. When inconsistencies occurred between toxicology results and self-reported use, positive toxicology findings were given priority, whereas self-reports were used to estimate frequency when toxicology was unavailable. Collateral information was considered when needed to clarify discrepancies.

For each substance, the number of use days during the previous month was estimated to derive a reliable measure of frequency of use.

All procedures were conducted in accordance with the Declaration of Helsinki and approved by the local Ethics Committee.

### 2.2. Measures

The diagnosis of psychiatric comorbidities and SUDs was established according to DSM-5-TR criteria.

To capture the multidimensional symptomatology of ADHD and associated emotional and functional impairments, a set of validated self-report and clinician-administered instruments was employed:

- The Adult ADHD Self-Report Screening Scale for DSM-5 (ASRS-5) is a six-item self-report scale assessing the frequency of core ADHD symptoms. Each item is rated on a 5-point Likert scale (0 = “never” to 4 = “very often”). A simple additive scoring (range: 0–24) was used, with higher scores indicating greater symptom burden. A score ≥ 14 was adopted as the cut-off for a positive ADHD screening [30].
- The Reactivity, Intensity, Polarity, and Stability Questionnaire (RIPoSt-40) is a 40-item self-report questionnaire designed to assess emotional dysregulation across four domains affective instability, negative emotionality, emotional impulsivity, and positive emotionality. In the present study, only the first three domains were included in the analyses, as the positive emotionality subscale was excluded because it is not directly related to the construct of emotional dysregulation, which was the focus of the investigation. The subscale scores were used to quantify the intensity and variability of emotional responses [31].
- The Clinical Global Impressions scale (CGI) is a clinician-rated instrument assessing the global severity of illness, taking into account symptoms, functioning, and psychosocial impairment [32].

Variables related to levomethadone dosing (baseline dose, maximum dose, dose change, and dose escalation indicator) and substance use frequency were operationalized as detailed in Table 1.

### 2.3. Statistical Analyses

Baseline characteristics are reported as medians and interquartile ranges for continuous variables and were compared using Mann–Whitney U tests, while categorical variables were compared using chi-square tests or Fisher’s exact test.

Longitudinal changes were analyzed using Linear Mixed Models (LMMs) estimated via Restricted Maximum Likelihood (REML), with time, ADHD screening group (ASRS ≥ 14 vs. <14), and levomethadone dose escalation (↑levo.) included as fixed effects, along with all two-way and three-way interactions among these factors. Random intercepts were specified for each subject, and an autoregressive covariance structure [AR(1)] was applied to model within-subject correlations across repeated measurements (baseline, T1, and T2; mean months = 2.13, SD = 0.64; and 6.20, SD = 0.91, respectively). This analytical approach is consistent with current best practices in longitudinal clinical research [33,34].

Post hoc pairwise comparisons were conducted on estimated marginal means (EMMEANS), with Bonferroni-adjusted *p*-values to control for multiple testing. The significance threshold was set at α = 0.05.

Linear mixed models included all participants with at least one valid follow-up per scale (see Table 1). Missing data due to loss at follow-up were handled under the Missing At Random (MAR) assumption, allowing for the inclusion of incomplete cases by using all available repeated measures.

For the ASRS scale, which was assessed only at two timepoints (baseline and T2), non-parametric Wilcoxon signed-rank tests were applied to evaluate within-subject changes over time.

All analyses were conducted using IBM SPSS Statistics (version 25.0; IBM Corp., Armonk, NY, USA).

## 3. Results

### 3.1. Baseline Sample Characteristics

A total of 83 participants completed the baseline assessment. As detailed in the Methods section, 71 had at least one valid follow-up for the clinical scales (RIPOST, CGI, and ASRS-5) and were included in the Linear Mixed Model (LMM) analyses. For substance use outcomes, baseline active users were: 30 for opioids, 34 for alcohol/sedatives, 38 for stimulants, and 26 for cannabis (THC); among these, 25, 28, 32, and 23 participants, respectively, had at least one valid follow-up and were included in the models (Table 1).

#### 3.1.1. Baseline Demographics and Clinical Features

Table 2 reports the baseline comparison between participants with and without ADHD-positive screening (ASRS ≥ 14 vs. <14). Individuals in the ADHD-positive group were significantly younger than their counterparts (Median = 43.5 vs. 50 years, *p* = 0.027) and were less likely to be employed (33.3% vs. 67.8%, *p* = 0.004).

The ADHD-positive subgroup showed a markedly higher prevalence of psychiatric comorbidities (87.5% vs. 40.7%, *p* < 0.001), family psychiatric history (66.7% vs. 27.1%, *p* < 0.001), and family history of suicide attempts (37.5% vs. 5.1%, *p* < 0.001).

Baseline scores on the CGI and RIPOST scales also reflected a more severe clinical profile: higher global illness severity (Median CGI = 5 vs. 4, *p* = 0.024) co-occurred with increased emotional impulsivity, affective instability, and negative emotionality (all *p* < 0.001).

Regarding substance use patterns, individuals screening positive for ADHD reported higher rates of concurrent alcohol and sedative use (58.3% vs. 33.9%, *p* = 0.040) and showed a trend toward increased stimulant use (62.5% vs. 39%, *p* = 0.051). No significant differences were observed for current opioid or cannabis (THC) use.

#### 3.1.2. Descriptive Statistics of Doses and Substance Use

Mean baseline levomethadone doses were similar across ADHD groups (≈38 mg), and the proportion of participants showing dose escalation (↑levomethadone) varied across the clinical scales and substance use domains (≈45–55%). The mean maximum Δmg among participants with at least one dose escalation ranged from +9 to +13 mg (Table 1).

### 3.2. Linear Mixed Model Results

#### 3.2.1. RIPOST Emotional Dysregulation

The LMM revealed a significant main effect of time for emotional impulsivity (F(2, 106.2) = 13.4, *p* < 0.001) and affective instability (F(2, 80.3) = 5.6, *p* = 0.005), with both dimensions showing a marked reduction already at T1 and remaining stable through T2. No significant changes were observed for negative emotionality (Table 3). In ASRS ≥ 14 group, estimated marginal means of emotional impulsivity decreased from 35.5 at baseline to 28.9 at T1 and 28.7 at T2, corresponding to a reduction of approximately 6–7 RIPOST points that was maintained over six months, whereas scores in ASRS < 14 participants remained essentially stable over time.

**Figure 1:**
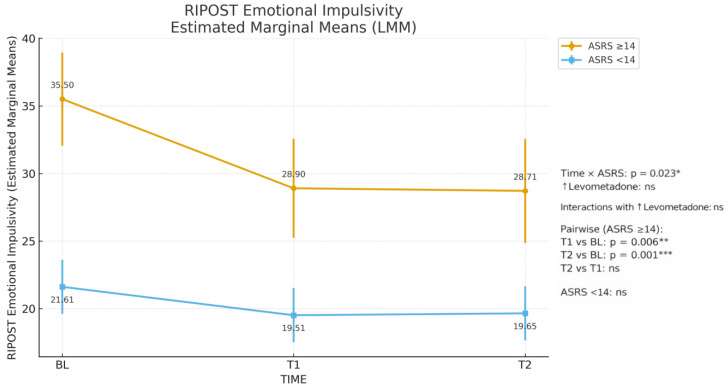
RIPOST Emotional Impulsivity Estimated Marginal Means (LMM). * *p* < 0.05; ** *p* < 0.01; *** *p* < 0.001; ns = not significant.

A strong main effect of ASRS ≥ 14 emerged across all subscales (all *p* ≤ 0.002), with higher scores in ADHD-positive screening participants. A significant time × ASRS interaction was observed for impulsivity (F(2, 106.2) = 3.9, *p* = 0.023), indicating that impulsivity decreased over time only in the ASRS ≥ 14 group, with a marked reduction already evident at T1 (T1 vs. BL, *p* = 0.006) and remaining stable thereafter (T2 vs. BL, *p* < 0.001; T2 vs. T1, n.s.; see Table 4 and Figure 1). Figure 1 visually illustrates this pattern, showing a clear downward shift in emotional impulsivity in the ASRS ≥ 14 group and a flat trajectory in the ASRS < 14 group. No effects involving dose escalation (↑levomethadone) reached significance.

#### 3.2.2. Clinical Global Impression (CGI)

A significant main effect of time was detected (F(2, 85.5) = 17.03, *p* < 0.001), with progressive reductions in CGI scores from baseline to both follow-ups. The greatest improvement occurred by T1 (Δ = −0.55 ***) and was further sustained at T2 (Δ = −0.70 ***).

A main effect of ASRS ≥ 14 group was also observed (F(1, 68.9) = 4.27, *p* = 0.025), with ADHD-positive participants showing higher clinical severity overall (Table 5).

No significant interactions involving dose escalation were detected.

#### 3.2.3. Substance Use Frequency

Among baseline active users, time significantly influenced alcohol/sedative (F(2, 22.7) = 6.20, *p* = 0.007 **), with a clear decline in days of use emerging at T1 and persisting through T2 (both *p* < 0.05 vs. baseline).

For opioid use, the effect of time was significant (F(2, 27.6) = 3.78, *p* = 0.035), but no pairwise contrast survived correction. This pattern indicates a modest overall reduction across the follow-up period, although no individual timepoint comparison reached statistical significance after adjustment for multiple testing. A main effect of dose escalation was observed only for stimulant use (F(1, 28.2) = 5.37, *p* = 0.028 *), indicating higher average stimulant use levels among participants with ↑levomethadone (Table 6).

No significant ASRS × time or three-way interactions emerged.

### 3.3. Additional and Exploratory Analyses

No significant ASRS × ↑levomethadone interactions were detected in the main models, suggesting that dose escalation did not differentially affect ASRS ≥ 14 vs. <14 participants.

When restricting the analysis to participants with screening positive for ADHD at baseline (ASRS ≥ 14), a paired-samples Wilcoxon test revealed a significant reduction in total ASRS scores from baseline to T2 (z = −2.3, *p* = 0.019), indicating an overall improvement in ADHD-related symptoms during levomethadone maintenance (Table 7).

## 4. Discussion

This observational study examined emotional, clinical, and substance use outcomes following the transition from racemic methadone (R,S-methadone) to levomethadone (R-methadone) in patients with opioid use disorder receiving methadone maintenance therapy, considering the moderating role of ADHD symptoms and dose escalation. The main finding was a significant reduction in emotional impulsivity over time, which emerged only among participants screening positive for ADHD (ASRS ≥ 14).

In contrast, emotional instability showed a significant decrease across the entire sample, regardless of ADHD screening status. The magnitude of this change was substantial, with ASRS ≥ 14 patients showing an average decrease of about 6–7 RIPOST points between baseline and follow-ups, while emotional impulsivity remained virtually unchanged in ASRS < 14 patients. This pattern suggests that levomethadone may exert a selective and clinically meaningful impact on impulsivity-related dimensions in individuals with prominent ADHD-related traits.

A plausible explanation for these findings lies in the pharmacodynamic profile of levomethadone. The R-enantiomer supports a more stable dopaminergic tone than the racemic formulation [19,35,36], whose S-enantiomer may attenuate dopaminergic signaling over time [24,37]. This pharmacological difference helps explain the selective improvement in impulsivity observed among participants with ADHD-related traits and is consistent with the absence of a dose–response gradient, as benefits emerged independently of dose escalation.

These results align with previous research showing that patients with ADHD exhibit greater emotional dysregulation within opioid-using populations [11], and the improvement in emotional control observed here supports the hypothesis that levomethadone contributes to affective stabilization, particularly in those with ADHD-related traits.

Clinical severity, as measured by the CGI, also improved significantly across time, reflecting a global stabilization of functioning and symptom burden. This effect was independent of ADHD status and dose escalation, suggesting a general clinical benefit of levomethadone maintenance in complex, comorbid patients [38,39].

Regarding substance use behavioral outcomes, days of use decreased mainly for alcohol/sedatives and opioids, while other substances remained relatively stable; this pattern suggests that the stabilizing effect of levomethadone on affective instability, impulsivity, and negative urgency likely contributed to reduced use of sedatives, alcohol, and opioids, substances typically associated with relief craving and emotional numbing [40,41,42,43].

Cannabis use did not show a clear reduction over time, and this finding is in line with evidence suggesting an interaction between ADHD and cannabinoid effects.

Some individuals with ADHD report using THC as a form of self-medication to reduce internal tension or restlessness; however, available evidence indicates that cannabis use does not yield a stable improvement in core ADHD symptoms and, particularly when chronic, may be associated with worsening of attention and executive functioning [44,45]. These cognitive effects are thought to arise from CB1-mediated alterations in prefrontal circuits regulating attention, working memory, and inhibitory control [46] that are not expected to be substantially modified by levomethadone’s dopaminergic stabilization.

In our cohort, ASRS scores improved over six months despite the stability of cannabis use, suggesting that the observed benefits were largely driven by levomethadone-related improvements in impulsivity and overall clinical stabilization rather than by changes in cannabis consumption.

Importantly, these analyses were restricted to baseline active users with at least one valid follow-up, ensuring that observed differences reflected true within-subject changes.

Furthermore, our baseline contingency analyses revealed significantly greater use of alcohol and sedatives, and a strong trend toward increased stimulant use, among participants with ASRS ≥ 14. This pattern is consistent with a dual relief-craving mechanism, where sedatives and alcohol are used to mitigate negative affect, while stimulants may be sought to counteract the impulsivity, low arousal, and executive dysfunction associated with ADHD.

Although dose escalation (↑levomethadone) did not emerge as a significant factor in predicting emotional or clinical improvement, ADHD-positive screening participants showed reductions in emotional impulsivity even at comparable doses, suggesting a higher sensitivity to the pharmacological effects of levomethadone rather than a dose–response gradient. This interpretation is clinically relevant: individuals with ADHD might benefit from the stabilizing properties of R-methadone. Our operationalization of dose adjustment as a binary variable (at least one increase during follow-up) was intended to capture a clinically meaningful need for re-titration in routine practice, providing a robust and easily interpretable indicator consistent with previous observational work examining dose increase versus maintenance strategies in opioid agonist treatments [28,33,38].

From a clinical perspective, these results emphasize the importance of monitoring impulsivity as a key domain of response to opioid agonist therapy. This aspect is especially relevant for dual-diagnosis patients, such as those with comorbid ADHD, where affective instability often interferes with treatment adherence and functional recovery. The findings also support the rationale for using levomethadone in patients who require both maintenance efficacy and emotional stabilization, as it may provide steadier dopaminergic modulation and improved affective control compared to racemic methadone. Accordingly, dose escalation should be guided by clinical severity and tolerability rather than used primarily as a strategy to manage emotional symptoms, since therapeutic benefits appear to emerge even at stable or moderate doses.

Systematic assessment of ADHD symptoms may help clinicians identify patients who are particularly likely to benefit from levomethadone rather than racemic methadone, especially when impulsivity and negative urgency are prominent. In such cases, choosing levomethadone as the agonist of choice and titrating the dose primarily according to clinical stabilization, rather than only to craving or withdrawal, could be considered as part of a personalized treatment strategy.

Furthermore, our data are consistent with a model in which levomethadone provides a dopaminergic “baseline stabilization” that could be complemented by ADHD-targeted pharmacotherapy. In clinical practice, combining levomethadone with stimulant medications such as methylphenidate or, when appropriate, non-stimulant agents might produce synergistic effects on executive functioning, emotional control, and craving regulation in dual-diagnosis patients. Although the present study did not formally test such combinations, the selective improvement observed in ASRS ≥ 14 individuals supports this hypothesis and warrants prospective controlled trials.

If replicated, these results could inform future guideline recommendations by encouraging routine ADHD screening in OUD programs and by supporting the preferential use of levomethadone, alone or in combination with ADHD pharmacotherapy, in patients with marked emotional impulsivity and affective dysregulation.

Several limitations should be considered. The sample size was modest, and attrition across follow-ups reduced the number of participants at T2, possibly limiting power to detect smaller effects. Moreover, as an observational study, the findings should be interpreted with caution, since this design does not allow for the level of experimental control and causal inference achievable in randomized controlled trials. Nevertheless, the real-world setting provides valuable ecological validity and reflects the complexity of routine clinical practice in opioid agonist therapy.

Urine toxicology testing was not performed with the same frequency across all participants, reflecting the constraints of real-world outpatient addiction services. To minimize the impact of this variability, toxicology results were complemented by structured self-reports and collateral information, consistent with harm-reduction monitoring approaches validated for alcohol and cocaine use. This integrated strategy helped ensure reliable estimation of substance use frequency despite heterogeneous testing schedules [47,48].

Future research should replicate these findings in larger samples and include neuropsychological or neurobiological markers to clarify the mechanisms linking levomethadone, emotional regulation, and dopaminergic sensitivity in ADHD. Further studies might also distinguish between reward and relief components of craving and test whether emotional improvements observed here are specific to levomethadone or generalize to other opioid agonist treatments.

## 5. Conclusions

This study provides preliminary evidence that transitioning from racemic methadone to levomethadone may offer specific benefits in emotional regulation and overall clinical stabilization among patients with opioid use disorder. Reductions in emotional impulsivity and clinical severity were observed independently of dose escalation, suggesting that these effects reflect the pharmacodynamic properties of the R-enantiomer rather than quantitative dose increases.

Patients screening positive for ADHD showed the greatest improvements, indicating that levomethadone may help address affective dysregulation and impulsive tendencies that often complicate treatment trajectories. Concurrent reductions in opioid, alcohol and sedative use support the possibility that enhanced emotional stability may reduce compensatory self-medication behaviors.

These findings underscore the value of recognizing ADHD symptoms in patients receiving treatment for opioid use disorder, as impulsivity appears to influence clinical course and response to care. Systematic screening may help identify subgroups who could benefit from more tailored interventions. Future studies should explore whether combining levomethadone with ADHD-specific pharmacotherapy enhances executive functioning, emotional regulation and relapse vulnerability.

Incorporating structured ADHD assessment into routine OUD pathways, and considering impulsivity profiles during treatment planning, may support a more individualized approach to care. Within this context, levomethadone may represent a suitable option for patients who show clinically relevant impulsivity and require stable agonist therapy.

## Figures and Tables

**Table 1:** Number of participants included in LMM analyses.

Scale/Substance	ASRS Group	*N* Included	↑Levo *n* (%)	Mean Δmg (Max Increase)	Baseline Levomethadone (mg, Mean ± SD)	*p*-Value (Baseline Dose)
Alcohol/Sedatives	<14	16	5 (31.3%)	6.50	37.81 ± 16.30	0.545
Alcohol/Sedatives	≥14	12	7 (58.3%)	9.57	41.91 ± 19.03	
CGI	<14	50	24 (48.0%)	9.90	38.70 ± 18.55	0.840
CGI	≥14	21	10 (47.6%)	11.45	38.95 ± 17.50	
Opioids	<14	20	13 (65.0%)	10.00	35.50 ± 14.06	0.864
Opioids	≥14	5	2 (40.0%)	8.75	35.00 ± 12.86	
RIPOST	<14	50	24 (48.0%)	9.90	38.70 ± 18.55	0.840
RIPOST	≥14	21	10 (47.6%)	11.45	38.95 ± 17.50	
Stimulants	<14	19	11 (57.9%)	9.09	38.94 ± 17.76	0.939
Stimulants	≥14	13	6 (46.2%)	13.75	38.07 ± 20.15	
THC	<14	13	5 (38.5%)	10.50	35.19 ± 14.80	0.779
THC	≥14	10	5 (50.0%)	10.50	32.50 ± 13.22	

RIPOST: Reactivity, Intensity, Polarity, and Stability questionnaire; ASRS: Adult ADHD Self-Report Scale; ↑Levo: levomethadone dose escalation. Mean Δmg (max increase) refers to the average of each patient’s maximum levomethadone increase from baseline among those with ↑Levo.

**Table 2:** Baseline Comparison Between ADHD Groups (ASRS < 14 vs. ASRS ≥ 14).

Variable	ASRS < 14 (*N* = 59) *N* (%)	ASRS ≥ 14 (*N* = 24) *N* (%)	χ^2^	*p* Value
Employed	40 (67.8%)	8 (33.3%)	8.309	0.004 **
Gender: female	6 (10.2%)	6 (25%)	Fisher’s Exact	0.096
Current opioid use	23 (39%)	7 (29.2%)	0.712	0.399
Current stimulant use	23 (39%)	15 (62.5%)	3.801	0.051
Current alcohol/sedative use	20 (33.9%)	14 (58.3%)	4.212	0.040 *
Current THC use	15 (25.4%)	11 (45.8%)	3.303	0.069
Psychiatric comorbidities	24 (40.7%)	21 (87.5%)	15.068	<0.001 ***
Organic/metabolic comorbidities	35 (59.3%)	17 (70.8%)	0.966	0.326
Family psychiatric history	16 (27.1%)	16 (66.7%)	11.263	<0.001 ***
Family history of suicide	5 (8.5%)	6 (25%)	Fisher’s Exact	0.070
Family history of suicide attempt	3 (5.1%)	9 (37.5%)	Fisher’s Exact	<0.001 ***
Current pharmacological treatment	35 (59.3%)	18 (75%)	1.817	0.178
**Variable**	**Median (IQR)**	**Median (IQR)**	**z**	***p***
Age (years)	50 (12)	43.50 (22.25)	−2.217	0.027 *
Baseline methadone dose (mg)	70 (50)	80 (37.50)	−0.363	0.717
Years on methadone	7 (10)	10 (10)	−0.524	0.600
RIPOST affective instability	29 (15)	51 (15.25)	−5.944	0.000 ***
RIPOST negative emotionality	30 (15)	43 (11.75)	−3.679	0.000 ***
RIPOST emotional impulsivity	21 (9)	36 (13.75)	−5.374	0.000 ***
CGI baseline	4 (2)	5 (1)	−2.25	0.024 *

* *p* < 0.05; ** *p* < 0.01; *** *p* < 0.001. RIPOST: Reactivity, Intensity, Polarity, and Stability questionnaire (RIPOST); CGI: Clinical Global Impressions scale; ASRS: Adult ADHD Self-Report Screening Scale. **Note**: Pearson’s Chi-square test was applied unless otherwise specified. Fisher’s Exact Test was used for variables with expected cell counts < 5.

**Table 3:** Fixed effects from Linear Mixed Models for RIPOST subscales.

Effect (LMM)	Emotional Impulsivity	Negative Emotionality	Affective Instability
Time	*p* < 0.001 *** T1–BL = −4.35 ** T2–BL = −4.37 *** T2–T1 = −0.02	*p* = 0.064	*p* = 0.005 ** T1–BL = −2.60 * T2–BL = −3.65 * T2–T1 = −1.05
ASRS ≥ 14 vs. <14	*p* < 0.001 *** Δ = +10.78 ***	*p* = 0.002 ** Δ = +8.10 **	*p* < 0.001 *** Δ = +17.85 ***
ASRS × Time	*p* = 0.023 * ≥14: T1–BL = −6.59 ** ≥14: T2–BL = −6.78 *** ≥14: T2–T1 = −0.18	*p* = 0.860	*p* = 0.249
↑Levo.	*p* = 0.552	*p* = 0.718	*p* = 0.630
↑Levo. × Time	*p* = 0.719	*p* = 0.780	*p* = 0.416
ASRS × ↑Levo.	*p* = 0.497	*p* = 0.840	*p* = 0.988
ASRS × ↑Levo. × Time	*p* = 0.623	*p* = 0.445	*p* = 0.968

* *p* < 0.05; ** *p* < 0.01; *** *p* < 0.001. RIPOST: Reactivity, Intensity, Polarity, and Stability questionnaire; ↑Levo.: levomethadone dose escalation; ASRS: Adult ADHD Self-Report Screening Scale. Note: “Δ” refers to estimated mean differences between each follow-up timepoint and baseline as derived from the Linear Mixed Model (LMM). **Note**: For the ASRS < 14 group, the ASRS × Time interaction was non-significant across all time comparisons (all *p* > 0.05).

**Table 4:** Estimated Marginal Means and Pairwise Comparisons for RIPOST Emotional Impulsivity (ASRS ≥ 14).

Time	Group	Estimated Mean	95% CI	Pairwise (*p*)
BL	ASRS ≥ 14	35.50	[32.06–38.93]	-
T1	ASRS ≥ 14	28.90	[25.24–32.56]	T1–BL *p* = 0.006 **
T2	ASRS ≥ 14	28.71	[24.87–32.56]	T2–BL *p* < 0.001 ***; T2–T1 *p* = 1.000

** *p* < 0.01; *** *p* < 0.001. ASRS: Adult ADHD Self-Report Screening Scale. T1 Mean months: 2.13 (0.65 SD); T2 Mean months: 6.2 (0.91 SD). No significant effects in the ASRS < 14 group.

**Table 5:** Fixed effects from LMM for CGI.

Effect (LMM)	CGI-Severity
Time	*p* < 0.001 *** T1–BL = −0.55 *** T2–BL = −0.70 *** T2–T1 = −0.15
ASRS ≥ 14 vs. <14	*p* = 0.025 * Δ = +0.57 *
ASRS × Time	*p* = 0.846
↑Levo.	*p* = 0.087
↑Levo. × Time	*p* = 0.553
ASRS × ↑Levo.	*p* = 0.819
ASRS × ↑Levo. × Time	*p* = 0.713

* *p* < 0.05; *** *p* < 0.001. CGI: Clinical Global Impressions scale; ↑Levo.: levomethadone dose escalation; ASRS: Adult ADHD Self-Report Screening Scale. Note: “Δ” refers to estimated mean differences between each follow-up timepoint and baseline as derived from the Linear Mixed Model (LMM).

**Table 6:** Fixed effects from LMM for Substance Use Frequency.

Effect (LMM)	Opioids	Stimulants	THC	Alcohol/ Sedatives
Time	*p* = 0.035 * T1–BL = −4.57 T2–BL = −5.31 T2–T1 = −0.74	*p* = 0.599	*p* = 0.454	*p* = 0.007 ** T1–BL = −2.24 * T2–BL = −3.68 * T2–T1 = −1.43
ASRS ≥ 14 vs. <14	*p* = 0.195	*p* = 0.213	*p* = 0.641	*p* = 0.582
ASRS × Time	*p* = 0.860	*p* = 0.812	*p* = 0.993	*p* = 0.065
↑Levo.	*p* = 0.851	*p* = 0.028 * Δ = +6.50 *	*p* = 0.187	*p* = 0.425
↑Levo. × Time	*p* = 0.578	*p* = 0.463	*p* = 0.083	*p* = 0.650
ASRS × ↑Levo.	*p* = 0.077	*p* = 0.297	*p* = 0.846	*p* = 0.212
ASRS × ↑Levo. × Time	*p* = 0.058	*p* = 0.299	*p* = 0.550	*p* = 0.889

* *p* < 0.05; ** *p* < 0.01. ↑Levo.: levomethadone dose escalation; ASRS: Adult ADHD Self-Report Screening Scale. **Note**. The main effect of time on opioid use was significant (*p* = 0 .035 *), although no pairwise comparisons reached significance after Bonferroni correction (all *p* > 0 .05). “Δ” refers to estimated mean differences between each follow-up timepoint and baseline as derived from the Linear Mixed Model (LMM).

**Table 7:** Wilcoxon ASRS total score.

Variable	Baseline *N* = 14 Median (IQR)	T2 *N* = 14 Median (IQR)	z	*p*
ASRS TOT (ASRS ≥ 14)	16.5 (4.5)	14 (5.5)	−2.3	0.019 *

* *p* < 0.05. ASRS: Adult ADHD Self-Report Screening Scale. **Note**: Analysis restricted to participants with baseline ASRS ≥ 14.

## Data Availability

The datasets generated and analyzed during the current study are not publicly available due to privacy restrictions but are available from the corresponding author on reasonable request.

## Abbreviations

The following abbreviations are used in this manuscript:

ADHD	Attention-Deficit/Hyperactivity Disorder
OUD	Opioid Use Disorder
ASRS-5	Adult ADHD Self-Report Scale for DSM-5
RIPOST	Reactivity, Intensity, Polarity and Stability Questionnaire
CGI	Clinical Global Impression
LMM	Linear Mixed Model
